# The conundrum of the connection between severe psychiatric disorders and dementia

**DOI:** 10.1590/1980-5764-DN-2024-0223

**Published:** 2025-04-07

**Authors:** Antonio Lucio Teixeira, Gabriel Alejandro de Erausquin, Rene L. Olvera

**Affiliations:** 1 University of Texas, Health Science Center at San Antonio, The Glenn Biggs Institute for Alzheimer's and Neurodegenerative Diseases, San Antonio TX, United States.; 2 University of Texas, Health Science Center at San Antonio, Long School of Medicine, Department of Neurology, San Antonio TX, United States.; 3 University of Texas, Health Science Center at San Antonio, Long School of Medicine, Department of Psychiatry and Behavioral Sciences, San Antonio TX, United States.

**Keywords:** Mental Health, Bipolar Disorder, Schizophrenia, Dementia, Alzheimer Disease, Frontotemporal Dementia, Saúde Mental, Transtorno Bipolar, Esquizofrenia, Demência, Doença de Alzheimer, Demência Frontotemporal

## Abstract

Psychiatric disorders have been recognized as important risk factors for neurodegenerative diseases, especially dementia. The strength of association varies among different psychiatric conditions, being more pronounced in severe mental illnesses, i.e., schizophrenia and bipolar disorder. Multiple mechanisms seem to underlie this association, such as high prevalence of cardiovascular and other physical morbidities, poor lifestyle choices, and accelerated aging, including ‘inflammaging’. They all represent opportunities for intervention, but it is still unclear whether current therapeutic approaches for psychiatric disorders can prevent the development of dementia. Other knowledge gaps include whether the risk of dementia applies to all patients with a certain condition, or if subgroups of patients are more vulnerable than others, whether different types of dementia are linked to specific psychiatric disorders.

## RESUMO.

Os transtornos psiquiátricos vêm sendo reconhecidos como importantes fatores de risco para doenças neurodegenerativas, especialmente as demências. A força de associção varia conforme as diferentes condições psiquiátricas, sendo mais pronunciada nos transtornos mentais graves, como esquizofrenia e transtorno bipolar. Múltiplos mecanismos parecem suportar essa associação, como a alta prevalência de morbidades cardiovasculares e outras, hábitos de vida, envelhecimento acelerado, incluindo ‘inflammaging’. Todos eles representam oportunidades para intervenção, mas ainda é incerto se abordagens terapêuticas dos transtornos psiquiátricos podem prevenir o desenvolvimento de demência. Outras lacunas do conhecimento envolvem definir se o risco de demência se aplica a todos pacientes com determinada condição ou se há subgrupos mais vulneráveis que outros, se diferentes tipos de demência estão ligados a transtornos psiquiátricos específicos.

## INTRODUCTION

Dementia is the final common state of many, often overlapping disease processes leading to loss of higher cognitive functions. Neurodegenerative diseases, especially Alzheimer's disease, are the main causes of dementia, followed by cerebrovascular diseases. With the aging population, the prevalence of dementia has significantly increased. It is estimated that, worldwide, over 150 million people will have dementia by 2050^1^.

In addition to older age, several factors have been identified as increasing the risk of dementia, including hypertension, hearing impairment, smoking, obesity, physical inactivity, diabetes, and excessive alcohol consumption^2^. More recently, in part because of the mental health impact of COVID-19 pandemic, psychiatric disorders have been in the spotlight as risk factors of dementia^3,4^.

This manuscript will review the current evidence implicating psychiatric disorders (focus on severe mental illnesses) as risk factors for dementia and discuss potential underlying mechanisms alongside the meaning of late-onset psychiatric disorders. Finally, we will highlight the multiple gaps in the literature on the complex association between severe psychiatric disorders and dementia.

### Psychiatric disorder as a risk factor for dementia: an overview

Clinical diagnosis of dementia relies primarily on the sequence in which language, memory, complex perceptual abilities, or the organization of social and motor behaviors are impaired by the progression of the underlying illness (Table 1). Conversely, etiological diagnosis depends on the identification of disease-specific biomarkers through different methods (e.g., cerebrospinal fluid measures, positron emission tomography neuroimaging, skin biopsy, etc.)^5^. Etiological diagnosis has become relevant with the recent development and availability of disease-modifying strategies, i.e., anti-amyloid antibodies^6^.

**Table 1:** Main neurocognitive domains affected in different stages of neurodegenerative diseases.

	Memory	Language	Behavior	Perception	Motor
Alzheimer's disease	Early	Early	Late	Late	Late or none
Frontotemporal degeneration	Late	Early	Early	Late	Late
Lewy body disease	Late	Late	Late	Early	Early

The term ‘psychiatric disorder’ encompasses a diverse group of conditions characterized by somewhat distinct combinations of behavioral and psychological symptoms that are individually difficult to distinguish from everyday life experiences. While neurodevelopmental issues, at least partially driven by genetic factors, play a major role in certain disorders (e.g., autism, intellectual disability), environmental stressors are pivotal in eliciting and/or altering progression of other ones (e.g., post-traumatic stress disorder, anxiety disorders, and depression). Severe mental illnesses, a term usually applied to schizophrenia and recurrent mood disorders, especially bipolar disorder (BD), emerge in the context of a complex interplay among genetic and environmental factors^7^.

Several psychiatric disorders have been linked to dementia. However, given their phenotypic and pathogenetic heterogeneity, it is important to define the individual risk of discrete conditions before assuming that all psychiatric disorders are risk factors for dementia. A recent British study using electronic health records on almost 1 million adults, and on 228,937 UK Biobank participants, found that a greater risk of dementia occurred with more severe psychiatric disorders^8^. The diagnoses of schizophrenia, BD and depression were associated with a subsequent diagnosis of dementia, with respective hazard ratios of 2.87 95% confidence interval — CI (2.7–3.04), 2.80 95%CI (2.6–3.1), 1.63 95%CI (1.6–1.7). The evidence for anxiety was mixed and consistent with former studies^9,10^. These distinct risk effects reflect, in part, meaningful ontological differences, i.e., they differ from a pathogenic perspective with very different contributions of biological and psychological factors. For instance, anxiety disorders are very common, encompass distinct but frequently overlapping conditions (e.g., panic episodes and generalized anxiety) that can be challenging to disentangle from daily life experiences where environmental stressors play a major precipitating and/or aggravating role^11^. These facts might explain the mixed results regarding the potential association between anxiety disorders and dementia. Conversely, depression has consistently been linked to dementia. However, the strength of association is lower than that observed for other recurrent mood disorders (i.e., BD), possibly reflecting the very heterogeneous clinical and biological nature of depression^12^.

Another relevant issue regarding the association between psychiatric disorders and dementia refers to the period of development of behavioral or psychiatric disorders. While early and/or mid-life psychiatric disorders are seen as risk factors, late-onset (in the sixth decade of life or later) disorders are frequently conceptualized as a prodrome of neurodegenerative disease^12^. Autism spectrum disorders and other neurodevelopmental conditions would fall under the first category. Also, several major psychiatric disorders, such as schizophrenia and mood disorders, emerge during adolescence and early adulthood. Chronic and/or recurrent psychiatric disorders are seen as risk factors for neurodegeneration later in life, and the mechanisms may involve progressive ‘wear and tear’ of body systems with increased allostatic load, pathological aggregation of proteins (e.g., beta amyloid, tau), and elusive disease-specific mechanisms^13^.

Late-onset behavioral syndromes can meet the DSM-5 criteria for psychiatric disorders. However, a significant proportion is subsyndromal even though the symptoms are a clear change from the person's baseline presentation and function. To account for the latter, the International Society to Advance Alzheimer's Research and Treatment (ISTAART) proposed ‘mild behavioral impairment’ (MBI) as a syndrome characterized by clinically meaningful behavioral symptoms affecting one of the five domains: motivation, affective regulation, impulse control, social cognition, and perception/thought content, that emerge after 50 years of age^14^. MBI has been conceptualized as the behavioral counterpart of mild cognitive impairment in the dementia spectrum.

Regarding underlying mechanisms, besides the ‘wear and tear’, it is worth mentioning the role played by cerebrovascular burden. Lifelong psychiatric disorders have increased frequency of cardiovascular morbidity and white matter disease, while cerebrovascular mechanisms contribute to the process of neurodegeneration. Cerebrovascular diseases (cortical and subcortical strokes, cerebral microvascular disease) can also trigger the development of late life psychiatric presentations (e.g., vascular depression and vascular dementia)^15^.

### Schizophrenia: from dementia praecox to neurodevelopmental disorder and back

The German neuropsychiatrist Emil Kraepelin (1856–1926) set the foundations of contemporary psychiatry nosology when proposing the distinction between *dementia praecox* (later called schizophrenia) and manic-depressive illness (currently, BD), the prototypes of severe mental illnesses. While both conditions can be challenging to differentiate in cross-sectional assessments (e.g., both can exhibit marked psychotic phenomena), Kraepelin emphasized their differential longitudinal course, with schizophrenia evolving with functional decline (8). As the illness primarily affects younger adults, the expression *dementia praecox* encapsulated this concept of progressive functional deterioration. Later, Eugen Bleuler (1857–1939) proposed the term schizophrenia — meaning ‘splitting of the mind’ — to replace *dementia praecox*, emphasizing the disintegration of the mind unit, as expressed by alogia, ambivalence, among other symptoms, in detriment of the related progressive course^16^.

The current conceptualization of schizophrenia acknowledges that cognitive deficits are among its core features alongside psychosis (i.e., hallucinations and delusions), and negative symptoms (i.e., blunted affect, abulia, alogia)^17^. Neurocognitive deficits in processing speed, attention, working memory, verbal and visual learning/memory, and social cognition are impaired in schizophrenia. As processing speed is the most affected domain and subserves other functions, some argue that cognitive deficits in schizophrenia are better defined as ‘generalized’ instead of domain-specific^17^. Conversely, others support the notion that distinct cognitive domains are affected by the disease process. One of the most robust pathophysiological findings in schizophrenia is increased subcortical, especially striatal, dopamine neurotransmission^18^. Dopamine, through their influence on fronto-striatal circuits, has been implicated in a series of cognitive functions, including attention, reward learning and decision-making^17,18^.

Schizophrenia-related cognitive deficits are established before the onset of psychotic symptoms during adolescence and/or early adulthood, indicating alterations in the neurodevelopmental trajectory of these patients, with their relatively stable course after the transition to the psychotic phase of the illnes^17,19,20^. While these facts contradict, at least in part, the historical view of schizophrenia as a dementing condition, there is evidence for a rapid decline in cognitive function later in life, especially over the age of 65, in individuals with schizophrenia^17,21,22^. Several studies have also demonstrated increased risk of dementia in people with schizophrenia with a relative risk around 2^10,23,24^. Interestingly, negative symptoms of schizophrenia, such as blunted affect, abulia/apathy and alogia, tend to worsen over time and phenotypically resemble late stages of dementing illnesses^25,26^. Therefore, cognitive trajectories in schizophrenia would be consistent with both neurodevelopmental and neurodegenerative patterns where an early cognitive decline created a vulnerability for dementia when exposed to future insults.

Independent processes have been implicated in the mechanisms underlying the increased risk of dementia in schizophrenia, such as low cognitive reserve, accelerated cognitive aging, medication exposure, increased frequency and/or severity of cardiovascular diseases^4,24^. For instance, patients with schizophrenia are at increased risk of cerebrovascular diseases and subsequent cognitive impairment and dementia^27^. Recent studies have also shown that schizophrenia and neurodegenerative diseases, including Parkinson's and Alzheimer's diseases, may share similar genetic architecture^28^. It remains to be determined whether schizophrenia is more vulnerable to a specific type of dementia and the pathophysiological mechanisms driving this association^29^. In addition to accelerated and/or intensified ‘wear and tear’ of body systems and cerebrovascular injury driven by schizophrenia, it is possible that disease-specific mechanisms play a role. For instance, impaired dopamine metabolism can lead to neurotoxicity through oxidative stress, neuroinflammation and apoptosis^30^.

### Bipolar disorder as a neuroprogressive condition

BD is characterized by a fluctuating course of mood symptoms between mania and depression poles with periods of euthymia^31^. It was originally described as a non-progressive condition within the Kraepelinian framework. However, at least a proportion of people with BD can exhibit cognitive deficits and evolve with deterioration of psychopathological and cognitive symptoms alongside functional decline, commonly referred in the literature as ‘neuro-progression’^32^.

Overall, the profile of cognitive symptoms in BD resembles the one observed in schizophrenia, with impairment of executive function and verbal memory but less severe^33^. There is also evidence of different cognitive subgroups (e.g., cognitively intact vs. selectively impaired vs. globally impaired) in BD, contrasting with a more homogeneous presentation in schizophrenia^34^. Cognitive symptoms are present across all mood phases, being more prominent during episodes of mania. Despite controversies around the concept of ‘neuro-progression’^35^, it has been acknowledged that people with BD may exhibit different longitudinal patterns of cognitive change, and a subgroup can evolve with cognitive deterioration^36^. The factors or clinical features of this subgroup of patients remain to be determined, and whether severity of presentation such as psychosis and/or schizoaffective disorder predict cognitive outcomes.

People with BD are also at increased risk of dementia, with an odds ratio around 2.3^3,10,37^. As for schizophrenia, it is uncertain whether BD is associated with specific types of dementia. However, some authors have suggested its relationship with behavioral variant of frontotemporal dementia (FTD)^38^. While cases of BD, especially of later onset, can represent a prodrome of FTD^39^, it is also possible that BD and FTD share similar genetic risk variants^40^. Regardless of these controversies, the symptomatic resemblance between features of mania/hypomania episodes and FTD is unequivocal, sometimes leading to a diagnostic challenge^41,42^.

While antipsychotics, valproate and other anticonvulsants have a negative influence on cognition, there is evidence that lithium can improve cognition, also exerting neuroprotective effects^43–45^. Given these effects of lithium, one question that emerges is whether it influences BD risk to dementia. Cross-sectional and registry studies have supported this assumption, but large controlled trials are needed to confirm it.

In addition to a shared genetic architecture with neurodegenerative diseases^46^, patients with BD have higher physical illness burden, including cardiovascular diseases^47^, enhanced inflammation with immune senescence^48–50^ and other accelerated aging mechanisms^51,52^. Altogether, these distinct mechanisms may contribute to neurodegenerative processes leading to the increased risk of cognitive impairment and dementia in BD^53^. Interestingly, studies evaluating blood and/or cerebrospinal fluid levels of amyloid and tau markers in patients with BD (and schizophrenia) did not show a biological signature compatible with Alzheimer's disease but confirmed an altered amyloid metabolism possibly related to disease-drive mechanisms, such as neuroinflammation^54,55^.

## DISCUSSION

Evidence, primarily from cross-sectional or case-control studies, has demonstrated the association between psychiatric disorders and dementia^3,4^. While this link may not be seen as a conundrum anymore, several gaps in knowledge and questions persist. The strength of association varies among different psychiatric conditions, being more pronounced in severe mental illnesses, i.e., schizophrenia and BD^8^. It seems that conditions with a more robust neurobiological substrate are at increased risk of dementia, indicating the possible involvement of disease-specific factors or mechanisms. For a better characterization of these disorder-related differences, more prospective or longitudinal studies involving community- and clinic-based people are warranted. It is also important to define whether the risk of dementia applies to all patients with a certain condition, or if subgroups of patients are more vulnerable than others. In the latter case, it would be relevant to identify vulnerability markers/factors for future personalized approaches. Besides the recognition of risk factors traditionally related to dementia (e.g., hypertension, diabetes, obesity, smoking) that are overrepresented in people with severe psychiatric disorders, it is important to map potential disease-specific ones that may not only predict dementia risk but also indicate targetable pathophysiological mechanisms.

Indeed, one of most puzzling issues refers to the neurobiological bases of dementia in the context of psychiatric disorders. It is possible that the processes underlying severe psychiatric disorders could directedly cause dementia and/or contribute to the pathological aggregation of proteins that define neurodegenerative diseases responsible for dementia. While disease-specific mechanisms remain elusive, general mechanisms related to the high prevalence of cardiovascular and other physical morbidities, poor lifestyle choices (e.g., sedentarism, smoking, drug use), stress-related ‘wear and tear’, and accelerated aging are more frequent and/or severe in people with severe psychiatric disorders^7^. They all represent open venues for investigation and, eventually, therapeutic targets. Promoting healthy lifestyle changes, such as regular physical activities and healthy diets, can have a positive impact in both mental health and dementia prevention^56,57^. Our group and others have investigated the potential acceleration of aging processes related to severe psychiatric disorders, including the aging role of chronic low-grade inflammation that characterizes both psychiatric disorders and dementia^49,51,52^. Aging mechanisms, including ‘inflammaging’, could be targets of intervention with potential benefits for both the psychiatric condition and cognitive decline. As aging is the major risk factor for neurodegenerative diseases, the increased risk of dementia in people with severe psychiatric disorders could be the consequence of this accelerated aging.

It also remains to be determined if different types of dementia (e.g., vascular vs. neurodegenerative; Alzheimer's vs. tauopathies vs. TDP-43 related vs. mixed) are linked to specific psychiatric disorders. As previously mentioned, at least from a clinical perspective, it is sometimes challenging to differentiate FTD from manic/hypomanic behaviors in protracted cases of BD^38,39^. It is also worth mentioning that psychiatric disorders have been linked to other neurodegenerative diseases, especially Parkinson's disease, but this area is less studied than dementia^58^.

A major unanswered question refers to whether current treatments of psychiatric disorders can prevent the development of dementia. There is some preliminary evidence supporting the role of lithium in BD^44^, but more definitive studies are needed. Regarding other therapeutic strategies, one major caveat is that most psychiatric treatments are essentially symptomatic and do not seem to change the trajectories of the illnesses or their underlying pathogenesis^59^. However, there is recent indirect evidence suggesting otherwise. In a community-based study involving 2,132 participants aged 55–85 years (mean age, 76 years), chronic and new anxiety were associated with increased risk of all-cause dementia^60^. The authors proposed that individuals with anxiety were more likely to engage in unhealthy lifestyle behaviors, such as poor diet and smoking, leading to cardiovascular diseases and, hence, dementia. Nevertheless, the resolved anxiety at follow-up significantly reduced the risk of dementia, suggesting that management of anxiety may be a prevention strategy for dementia^60^. Antidepressant treatment of late-life depression improves cognition, specifically memory and learning, raising the possibility of preventing further cognitive decline^61^.

In sum, there are several knowledge gaps regarding the association between psychiatric disorders and risk of dementia. Searching for the related answers may advance understanding of the pathophysiology of mental illnesses, their therapeutic management and, ultimately, contribute to dementia prevention.
